# Sociodemographic Trends in Daily, Weekly and Less Than Weekly Cannabis Use in Australia

**DOI:** 10.1111/dar.70207

**Published:** 2026-07-27

**Authors:** Janni Leung, Gary Chung Kai Chan, Ruofan Zhang, Wayne D. Hall, Caitlin McClure‐Thomas

**Affiliations:** ^1^ National Centre for Youth Substance Use Research, School of Psychology The University of Queensland Brisbane Australia

## Abstract

**Introduction:**

In the Australian 2001–2013 National Drug Strategy Household Surveys (NDSHS), frequent cannabis use declined among higher socioeconomic groups while remaining stable among lower socioeconomic groups, suggesting a widening socio‐demographic gap. This study examined trends in cannabis use frequency and sociodemographic disparities in Australia up to 2022/23.

**Methods:**

Data were from eight waves of the NDSHS between 2001–2022/23 (total *N* = 197,484). Past 12‐month cannabis use was categorised as daily, weekly, less than weekly and no use. Multinomial regression models assessed associations between cannabis use, year and sociodemographic factors. Interaction terms examined changes in disparities over time.

**Results:**

Daily cannabis use ranged from 1.25% [95% confidence interval 1.08–1.45] to 1.67% [1.41–1.98] between 2001 and 2019, then increased to 2.17% [1.92–2.46] in 2022/23. Weekly and less frequent use remained stable. More frequent use was observed among males, younger adults, individuals living in outer regional or rural areas, unpartnered individuals, those with lower educational attainment, and those from more socioeconomically disadvantaged areas. Year‐by‐age interactions indicated larger increases in daily use among middle‐aged and older adults. No interactions were observed between survey year and other socioeconomic indicators. The increase in daily use was attenuated in sensitivity analyses excluding medicinal and prescribed cannabis use.

**Discussion and Conclusions:**

Daily cannabis use in Australia increased in 2022/23, with a smaller effect if we exclude medicinal use, while other patterns of use remained unchanged. Sociodemographic disparities remained. Continued monitoring is needed to determine whether this represents a sustained trend and to address underlying drivers of inequalities.

## 1 Introduction

The majority of epidemiological studies on cannabis use have examined it dichotomously (e.g., any use vs. no use). It is also important to examine differences in the frequency and intensity of use because daily or near‐daily cannabis use is associated with greater risks of dependence and adverse health outcomes [1]. Sociodemographic differences in frequent cannabis use may disproportionately affect already disadvantaged groups. Understanding how these disparities evolve over time is particularly important in the context of changing cannabis policies and increasing access to cannabis through medical legalisation. In Australia, medicinal cannabis was legalised in 2016, and in 2020 the Australian Capital Territory permitted adults to possess and cultivate small amounts for personal use. Evidence from Australia also suggests increasing cannabis use and intentions since 2013, particularly among middle‐aged and older adults, alongside more liberal attitudes toward cannabis [2, 3].

A previous study examined socioeconomic differences in cannabis use trends using nationally representative data from the 2001–2013 National Drug Strategy Household Surveys (NDSHS) in Australia [4]. It found significant socioeconomic differences in cannabis use patterns over time, with frequent cannabis use (daily and weekly) declining at a faster rate among higher than lower socioeconomic groups. Individuals with higher education showed reductions in daily use (1.4% to 0.9%, *p* < 0.001) and weekly use (2.5% to 1.7%, *p* < 0.001), and those with middle and higher incomes showed reductions in weekly use (3.2% to 2.3%, *p* = 0.004 and 2.1% to 1.3%, *p* = 0.005, respectively). By contrast, cannabis use frequency remained stable among individuals with lower income and/or education.

Since that study, the cannabis policy environment and social attitudes toward cannabis have continued to evolve in Australia. Updated analyses are therefore warranted to examine whether patterns of cannabis use frequency and related sociodemographic disparities have changed in more recent years. In this study, we updated these analyses using NDSHS data from 2001 to 2022/23 to examine sociodemographic differences in cannabis use frequency over time, with particular focus on daily use given its stronger association with cannabis dependence and other adverse health outcomes.

## 2 Methods

### 2.1 Data Sources

We analysed data from the Australian NDSHS, a national household survey conducted in 2001 (*N* = 26,744), 2004 (*N* = 29,445), 2007 (*N* = 23,365), 2010 (*N* = 26,648), 2013 (*N* = 23,855), 2016 (*N* = 23,749), 2019 (*N* = 22,015) and 2022–2023 (*N* = 21,663) [5]. The total sample size in the appended dataset from 2001–2023 was 197,484. The NDSHS is a nationally representative survey of Australians aged 14 years and older that measures variables related to participants' use of alcohol, tobacco and other drugs, including cannabis.

### 2.2 Measures

Frequency of cannabis use was categorised as follows: daily use, weekly (but less than daily) use, less than weekly use, and no use in the past 12 months. This measured any cannabis use because the surveys before 2019 did not separately measure the frequency of non‐medical and medical use. The sociodemographic variables included: sex, age groups, area of residence (major cities, inner regional, outer regional/remote), marital status, employment status, education and Socio‐Economic Indexes for Areas (SEIFA) quintiles. SEIFA is a measure of relative socioeconomic advantage and disadvantage based on participants' area of residence.

### 2.3 Data Analysis

Level of missing data for cannabis use was low (0.9%–3.6%), and the variables that accounted for the most missing data were marital status, employment status and education levels (0.4%–8.6%; Table S1). To reduce bias due to missing data, we used multiple imputation via chained equations, with distributions remaining consistent post‐imputation (Table S2). Results presented were based on the imputed dataset and were conducted in Stata, applying survey weights, primary sampling unit (psu), and strata to account for the complex survey design.

We estimated, in each survey year, the prevalence of daily, weekly (but less than daily), and less‐than‐weekly cannabis use with 95% confidence intervals (CI). Associations with socio‐demographic variables were assessed using multinomial regression. Model 1 included the main effects of year and the socio‐demographic predictors, and model 2 included the variables included in model 1 plus their interaction terms with survey year. There were no collinearity issues, with pairwise spearman correlations among explanatory variables all below 0.70, and all variables were entered into the model together. Adjusted odds ratios (OR) and 95% CIs were estimated using no use as the reference category. Given the observed increase in daily cannabis use in recent years, the legalisation of medical cannabis in Australia in 2016, and that the NDSHS asked if cannabis was prescribed or used for medicinal reasons in the 2019 and 2022/23 surveys, we conducted two post hoc sensitivity analyses to examine the effect of year on daily cannabis use: (i) after excluding participants who reported using cannabis for medicinal purposes only (*n* = 398); and (ii) after excluding participants who reported that they always used medical cannabis that was prescribed (*n* = 148).

## 3 Results

### 3.1 Participants Characteristics

Survey participants were approximately balanced in sex, age and SEIFA; 72.8% [72.5%–73.1%] lived in major cities, 59.8% [59.4%–60.2%] were married/de facto, 58.0% [57.7%–58.4%] were employed, and 65.0% [64.6%–65.3%] had high school education or above (Table S2).

Across all years overall, 89.5% [89.3%–89.7%] of participants did not report using cannabis in the past year, 6.7% [6.5%–6.9%] reported using it less than weekly, 2.3% [2.1%–2.4%] used weekly (but less than daily) and 1.5% [1.5%–1.6%] used daily.

### 3.2 Frequency of Cannabis Use by Year

**FIGURE 1:**
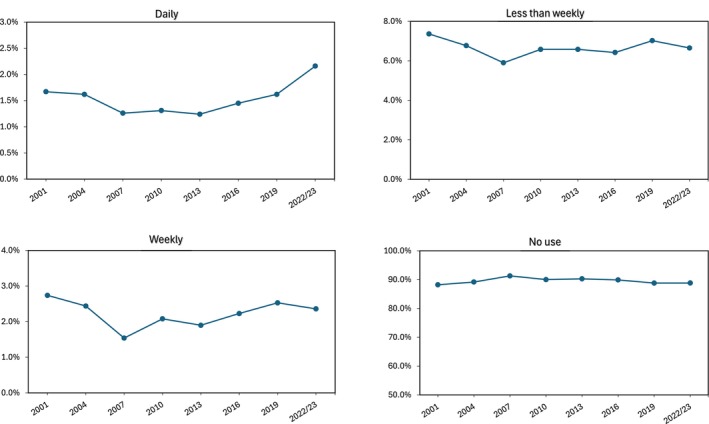
Weighted prevalence of daily, weekly (but less than daily), less than weekly and no cannabis use by year from 2001–2023. *Note:* Data table and 95% confidence intervals are available in Table S3.

Between 2001 and 2019, the prevalence of daily cannabis use varied between 1.25% [1.08%–1.45%] to 1.67% [1.41%–1.98%]. It increased to 2.17% [1.92%–2.46%] in 2022/23 (Figure 1 and Table S3). There were no substantial changes in weekly or less than weekly cannabis use, as indicated by the overlapping confidence intervals.

### 3.3 Cannabis Use by Sociodemographic Factors

Descriptive statistics from crosstabulation of cannabis use frequency by sociodemographic factors showed that cannabis use was more common among males, younger adults, people living in outer regional or rural areas, those who were not partnered, those who were unemployed, individuals with lower educational attainment, and those living in more socioeconomically disadvantaged areas (data in Table S4).

Multinomial regression results from the main effects model are in Table 1. Corresponding to trends observed in Figure 1, compared with 2001, the odds of cannabis use were generally lower in earlier survey years between 2004 and 2016, but daily use increased in 2022/23 (OR = 1.43 [1.25–1.64]). The sensitivity analysis comparing the effect of year in 2022/23 with 2019 showed higher odds of daily cannabis use in 2022–23 in both datasets that either included (OR = 4.43 [2.28–8.61]) and excluded (OR = 3.42 [1.59–7.36]) individuals who reported using cannabis only for medicinal reasons, or excluded individuals who reported that they always used medicinal cannabis that was prescribed (OR = 2.86 [1.08–6.48]), with a reduced effect size.

**TABLE 1:** Multinomial regression on cannabis use by year and socio‐demographic factors in the National Drug Strategy Household Surveys 2001–2022/23.

Main effects	Adjusted odds ratios (ref = no use)					
	Daily use (*n* = 2962) vs. no use (*n* = 176,748)		Weekly but less than daily (*n* = 4542) vs. no use (*n* = 176,748)		Less than weekly (*n* = 13,232) vs. no use (*n* = 176,748)	
	OR [95% CI]	*p*	OR [95% CI]	*p*	OR [95% CI]	*p*
Year (ref = 2001)						
2004	0.90 [0.80–1.02]	0.114	0.84 [0.76–0.93]	0.001	0.82 [0.77–0.88]	< 0.001
2007	0.71 [0.61–0.82]	< 0.001	0.58 [0.51–0.66]	< 0.001	0.68 [0.63–0.74]	< 0.001
2010	0.66 [0.57–0.76]	< 0.001	0.72 [0.64–0.81]	< 0.001	0.80 [0.74–0.85]	< 0.001
2013	0.72 [0.62–0.84]	< 0.001	0.68 [0.60–0.77]	< 0.001	0.83 [0.77–0.90]	< 0.001
2016	0.82 [0.72–0.95]	0.007	0.74 [0.66–0.83]	< 0.001	0.85 [0.79–0.91]	< 0.001
2019	0.96 [0.83–1.10]	0.560	0.84 [0.75–0.94]	0.004	0.93 [0.87–1.00]	0.066
2022/23	1.43 [1.25–1.64]	< 0.001	0.96 [0.85–1.08]	0.483	0.92 [0.85–0.99]	0.027
Sex (ref = female)						
Male	2.34 [2.17–2.52]	< 0.001	2.39 [2.24–2.56]	< 0.001	1.45 [1.39–1.50]	< 0.001
Age, years (ref = 40–50)						
12–17	0.13 [0.10–0.17]	< 0.001	0.36 [0.31–0.42]	< 0.001	1.04 [0.95–1.15]	0.405
18–24	1.37 [1.21–1.55]	< 0.001	1.54 [1.38–1.71]	< 0.001	2.38 [2.23–2.55]	< 0.001
25–29	1.75 [1.54–1.98]	< 0.001	1.62 [1.45–1.81]	< 0.001	2.26 [2.11–2.42]	< 0.001
30–39	1.33 [1.19–1.48]	< 0.001	1.36 [1.24–1.50]	< 0.001	1.49 [1.41–1.59]	< 0.001
50–59	0.52 [0.46–0.59]	< 0.001	0.63 [0.56–0.70]	< 0.001	0.55 [0.51–0.59]	< 0.001
60+	0.08 [0.07–0.10]	< 0.001	0.13 [0.11–0.15]	< 0.001	0.14 [0.12–0.15]	< 0.001
Area of residence (ref = major cities)						
Inner regional	1.20 [1.09–1.33]	< 0.001	1.19 [1.09–1.29]	< 0.001	1.10 [1.04–1.16]	0.001
Outer regional or rural	1.46 [1.32–1.61]	< 0.001	1.48 [1.36–1.62]	< 0.001	1.20 [1.13–1.27]	< 0.001
Marital status (ref = married/de facto)						
Not partnered	2.62 [2.42–2.85]	< 0.001	2.77 [2.58–2.97]	< 0.001	2.16 [2.07–2.26]	< 0.001
Employment status (ref = employed)						
Not employed	1.45 [1.33–1.57]	< 0.001	1.10 [1.02–1.18]	0.009	0.80 [0.76–0.84]	< 0.001
Education (ref = completed high school)						
No high school	1.85 [1.71–2.01]	< 0.001	1.27 [1.18–1.37]	< 0.001	0.86 [0.82–0.90]	< 0.001
SEIFA quintile (ref = highest)						
Lowest	1.76 [1.55–1.99]	< 0.001	1.26 [1.14–1.40]	< 0.001	0.82 [0.77–0.88]	< 0.001
2nd	1.50 [1.32–1.70]	< 0.001	1.10 [0.99–1.22]	0.076	0.82 [0.77–0.87]	< 0.001
3rd	1.35 [1.19–1.53]	< 0.001	1.15 [1.04–1.27]	0.007	0.89 [0.84–0.95]	< 0.001
4th	1.11 [0.97–1.26]	0.124	1.08 [0.98–1.19]	0.124	0.89 [0.85–0.95]	< 0.001

*Note:* All variables were entered into the model together and ORs presented are adjusted odds ratios. A *p*‐value of < 0.001 was used to indicate significance, which results meeting the cut‐off are indicated in bold. Abbreviations: CI, confidence interval; OR, odds ratio; SEIFA, Socio‐Economic Indexes for Areas.

The multinomial regression also showed that males had significantly higher odds of cannabis use across all frequencies compared with females, including more than twice the odds of daily use (OR = 2.34 [2.17–2.52]) and weekly use (OR = 2.39 [2.24–2.56]), and higher odds of less than weekly use (OR = 1.45 [1.39–1.50]). Age was strongly associated with cannabis use. Compared with adults aged 40–49 years, individuals aged 25–29 had the highest odds of daily use (OR = 1.75 [1.54–1.98]), followed by those aged 18–24 (OR = 1.37 [1.21–1.55]). Individuals living outside major cities had higher odds of cannabis use than those living in major cities, with those in outer regional or rural areas having 1.46 [1.32–1.61] times higher odds of daily use and 1.48 [1.36–1.62] times higher odds of weekly use. Unpartnered individuals had markedly higher odds of cannabis use across all frequencies compared with those who were married or in de facto relationships, including 2.62 [2.42–2.85] times higher odds of daily use. Being unemployed was associated with higher odds of daily use (OR = 1.45 [1.33–1.57]). Lower educational attainment was associated with higher odds of daily (OR = 1.85 [1.71–2.01]) and weekly use (OR = 1.27 [1.18–1.37]). Individuals in the lower SEIFA quintiles had higher odds of daily use (lowest SEIFA OR = 1.76 [1.55–1.99], 2nd OR = 1.50 [1.32–1.70], 3rd OR = 1.35 [1.19–1.53]). Those in the lowest SEIFA also had higher odds of weekly use (OR = 1.26 [1.14–1.40]). However, there were lower odds of less than weekly use among lower SEIFA quintiles (Table 1).

**FIGURE 2:**
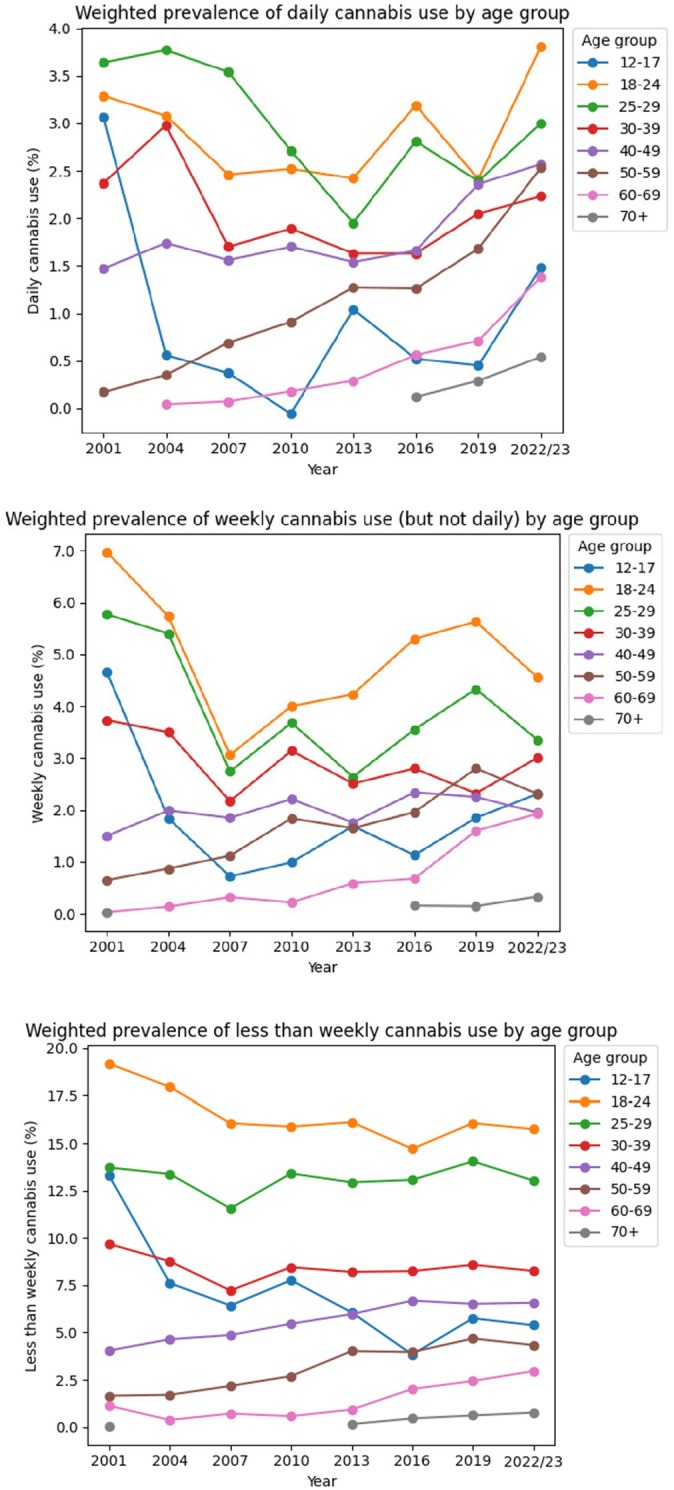
Weighted prevalence of daily, weekly (but less than daily), and less than weekly cannabis use by year from 2001–2023 by age groups. *Note:* Data table and 95% confidence intervals are available in Table S6.

There were trends of year by age interactions (Table S5). The association between younger age and higher odds of daily or weekly cannabis use was weaker in 2022/23, but stronger in the older age groups. Figure 2 shows that young adults aged 18–24 and 25–29 consistently had the highest prevalence across all frequencies of cannabis use, with levels declining in the 2010s then daily use increased in 2022/23. In contrast, older adults showed an upward trend over time. These findings suggest a shift toward increasing cannabis use among middle‐aged and older adults.

## 4 Discussion

We found that daily cannabis use in Australia ranged from 1.25% to 1.67% between 2001 and 2019 before increasing to 2.2% in 2022/23. The attenuated increase after excluding people who used cannabis for medicinal reasons and those who reported only using prescribed medicinal cannabis suggests that changes in access to medicinal cannabis may have contributed to the observed rise in daily use. Our finding that daily cannabis use increased warrants attention, as daily use is associated with higher risks of dependence and other adverse health outcomes [6]. This finding may suggest that the frequency of cannabis use increased among Australian adults who already used cannabis, which is a pattern that has also been observed in the USA [7, 8].

Consistent with previous research, we identified persistent socio‐demographic differences in the frequency of cannabis use [4, 9, 10]. High risk subpopulation groups included those aged 18–39, males, individuals in outer regional/rural areas, unpartnered people, unemployment, those with lower educational attainment, and people from more disadvantaged areas. These risk factors have important public health implications. For instance, the concentration of daily cannabis use among people living in lower SEIFA areas, together with evidence that frequent cannabis use is more strongly associated with cannabis‐related harms [1], including cannabis use disorder [6], suggests a need for prevention and support strategies that are responsive to socioeconomic disadvantage.

Our findings differ somewhat from a previous analysis of the 2001–2013 NDSHS, which found that frequent cannabis use declined among higher socioeconomic groups while remaining stable among lower socioeconomic groups, resulting in a greater concentration of regular use among disadvantaged populations [4]. In the current study, we found no consistent year‐by‐SEIFA interactions when we expanded the analysis to include datasets between 2001–2022/23. It may be that the social gradient in cannabis use frequency has been reduced in recent years. Our findings suggest that daily use rose in recent years and that socioeconomic disparities persisted.

In addition, our study found significant year‐by‐age interactions in cannabis use. While the overall prevalence of daily cannabis use remained highest among adults aged 18–39, increases in daily use were more consistently observed among middle‐aged and older adults. This is consistent with previous research showing increasing positive attitudes and cannabis use in older cohorts in comparison to younger cohorts, and similar trends have been observed in the USA and may reflect, in part, the use of cannabis for medical reasons among older adults [2, 11].

Increases in daily cannabis use in 2022/23 may partly reflect greater accessibility and legal availability, including the federal legalisation of medicinal cannabis and the 2020 ACT personal use reforms. Cannabis advertising to the public is not permitted in Australia; however, there have been breaches and their promotion has been documented [12]. Given evidence that exposure to cannabis advertising is positively associated with cannabis use [13], these findings highlight the importance of actively monitoring and enforcing restrictions on cannabis promotion, rather than relying on legislation alone.

Although our sensitivity analysis suggested that the increase was not explained solely by individuals reporting medicinal cannabis use, previous studies suggest that some people access medicinal cannabis for recreational purposes [14]. Second, public attitudes have shifted, with declining perceptions of harm and rising support for legalisation in Australia [2, 15]. These patterns mirror findings from the U.S. where an increased prevalence of cannabis use partly explains growing support for legalisation [16].

Public health approaches are needed to inform Australians about the health risks associated with high frequency cannabis use, particularly daily use. Evidence‐based resources, such as the Lower Risk Cannabis Use Guidelines [17] and patient information provided by the Therapeutic Goods Administration [18] could be more widely promoted to inform the public about the limited evidence supporting many medical uses of cannabis [19].

### 4.1 Limitations

This study relied on self‐reported data and so cannabis use may be underreported. Because the NDSHS data are cross‐sectional, causal inference cannot be made [20]. Therefore, although the increase in daily cannabis use between 2019 and 2022/23 was attenuated after excluding people who reported prescribed medicinal cannabis use, we cannot determine whether these individuals would have otherwise increased their use in the absence of access to prescribed cannabis. Future studies using longitudinal data or quasi‐experimental designs that compare jurisdictions with different cannabis policy settings could help clarify whether changes in medicinal cannabis access have contributed to trends in daily cannabis use. The NDSHS is a household survey, so it underrepresents high‐risk populations, such as homeless individuals and those residing in institutional settings. Although rates of missing data were still low, there was a higher proportion of missing data for cannabis use frequency in 2022/23 (3.6% vs. 1.3% in 2019). This may be a random fluctuation, or it may reflect increased reluctance to answer this item. This could affect prevalence estimates. Under‐reporting of substance use behaviours has been noted previously in Australia, such as for methamphetamine use [21]. Future research could explore whether rising non‐response reflects changing attitudes toward frequent cannabis use.

The timing of data collection may not fully align across survey years because fieldwork for the 2022/23 NDSHS spanned two years. This may affect cannabis use estimates due to potential period effects. Although fieldwork periods were largely comparable across survey waves, future research should continue to monitor trends in daily cannabis use in the NDSHS and triangulate findings with other datasets and study designs to strengthen robustness.

## 5 Conclusion

Daily cannabis use in Australia fluctuated between 2001 and 2019, then a small increase was observed in 2022/23. There were persistent sociodemographic disparities in cannabis use, with daily use more common among males, younger adults, socioeconomically disadvantaged groups, and people living outside major cities. Evidence suggested that the increase in daily cannabis use was apparent, including in people across different socioeconomic status groups. There were indications that daily use has increased more among middle‐aged and older adults in recent years. Given that daily cannabis use is more strongly associated with cannabis use disorders and other adverse health outcomes, these trends highlight the importance of continued monitoring of cannabis use patterns in Australia to establish whether this represents a sustained trend and to better understand the underlying drivers. Public health strategies are needed to communicate the risks associated with high‐frequency cannabis use.

## Author Contributions

Conception and design: Wayne D. Hall, Janni Leung, Gary Chung Kai Chan. Analysis: Janni Leung, Gary Chung Kai Chan, Ruofan Zhang. Interpretation: All authors. First draft: Janni Leung, Caitlin McClure‐Thomas. Subsequent drafts: All authors. Final approval and accountability of the work: All authors.

## Funding

The authors have nothing to report.

## Conflicts of Interest

The authors declare no conflicts of interest.

## Supporting information


**Table S1:** Levels of missing data in the NDSHSs 2001–2022‐23 across variables of interest.
**Table S2:** Weighted frequency of cannabis use and participant characteristics in the NDSHS 2001–2022/23 combined (*N* = 197,484).
**Table S3:** Data table for weighted frequency of cannabis use by year in the NDSHS 2001–2022/23 (*n* = 197,484).
**Table S4:** Prevalence of cannabis use by sociodemographic factors in the NDSHS 2001–2022/23 combined (*N* = 197,484).
**Table S5:** Significant year interactions from the multinomial regression on cannabis use.
**Table S6:** Prevalence of cannabis use by age in the NDSHS 2001–2022/23.

## Data Availability

The data that support the findings of this study are available on request from the corresponding author. The data are not publicly available due to privacy or ethical restrictions.
