# Supplementary material for: Sociodemographic Trends in Daily, Weekly and Less Than Weekly Cannabis Use in Australia
Source: Drug Alcohol Rev. 2026 Jul 27;45(6):e70207. doi: 10.1111/dar.70207 (PMC13407215; doi:10.1111/dar.70207)
Supplement: Supplementary file 1 — Table S1: Levels of missing data in the NDSHSs 2001–2022‐23 across variables of interest. Table S2: Weighted frequency of cannabis use and participant characteristics in the NDSHS 2001–2022/23 combined (N = 197,484). Table S3: Data table for weighted frequency of cannabis use by year in the NDSHS 2001–2022/23 (n = 197,484). Table S4: Prevalence of cannabis use by sociodemographic factors in the NDSHS 2001–2022/23 combined (N = 197,484). Table S5: Significant year interactions from the multinomial regression on cannabis use. Table S6: Prevalence of cannabis use by age in the NDSHS 2001–2022/23. [file DAR-45-0-s001.docx]

**Supplementary Materials**

**Sociodemographic trends in the frequency of cannabis use in Australia**

**Table S1. Levels of missing data in the NDSHSs 2001-2022-23 across variables of interest**

| **Variables** | **Missing data** | | | | | | | |
| --- | --- | --- | --- | --- | --- | --- | --- | --- |
|  | **2001** | | **2004** | | **2007** | | **2010** | |
|  | **N=26744** | | **N=29445** | | **N=23365** | | **N=26648** | |
|  | **n** | **%** | **n** | **%** | **n** | **%** | **n** | **%** |
| Cannabis use frequency | 342 | 1.3% | 277 | 0.9% | 315 | 1.4% | 409 | 1.5% |
| Sex | 0 | 0.0% | 0 | 0.0% | 0 | 0.0% | 0 | 0.0% |
| Age | 0 | 0.0% | 0 | 0.0% | 0 | 0.0% | 0 | 0.0% |
| Area of residence | 0 | 0.0% | 0 | 0.0% | 28 | 0.1% | 0 | 0.0% |
| Marital status | 118 | 0.4% | 593 | 2.0% | 814 | 3.5% | 1,495 | 5.6% |
| Employment status | 736 | 2.8% | 856 | 2.9% | 1205 | 5.2% | 2,301 | 8.6% |
| Education | 0 | 0.0% | 1 | 0.0% | 27 | 0.1% | 41 | 0.2% |
| SEIFA quintile | 0 | 0.0% | 0 | 0.0% | 0 | 0.0% | 0 | 0.0% |
|  | **2013** | | **2016** | | **2019** | | **2022/23** | |
|  | **N=23855** | | **N=23749** | | **N=22015** | | **N=21663** | |
|  | **n** | **%** | **n** | **%** | **n** | **%** | **n** | **%** |
| Cannabis use frequency | 408 | 1.7% | 243 | 1.0% | 295 | 1.3% | 784 | 3.6% |
| Sex | 0 | 0.0% | 0 | 0.0% | 0 | 0.0% | 524 | 2.4% |
| Age | 0 | 0.0% | 0 | 0.0% | 0 | 0.0% | 0 | 0.0% |
| Area of residence | 0 | 0.0% | 0 | 0.0% | 0 | 0.0% | 0 | 0.0% |
| Marital status | 599 | 2.5% | 560 | 2.4% | 99 | 0.4% | 501 | 2.3% |
| Employment status | 1,495 | 6.3% | 1227 | 5.2% | 1112 | 5.1% | 1401 | 6.5% |
| Education | 890 | 3.7% | 672 | 2.8% | 809 | 3.7% | 1289 | 6.0% |
| SEIFA quintile | 0 | 0.0% | 0 | 0.0% | 0 | 0.0% | 0 | 0.0% |

To reduce potential bias due to missing data, we ran multiple imputations using chained equations. We generated five imputed datasets using sequential regression techniques. We applied predictive mean matching with five nearest neighbors. All variables with missing values were included in the imputation model as both outcomes and predictors, while complete variables (year, age group, area of residence, and socioeconomic status) served as additional predictors. The imputation approach preserved relationships between variables while accounting for uncertainty in the missing values. Levels of missing data for all variables met the low level of missing data threshold of <5% (Table S1), except for marital, employment and education status which had up to 8.6% of missing data.

**Table S2. Weighted frequency of cannabis use and participant characteristics in the NDSHS 2001-2022/23 combined (N=197,484)**

|  | | Original data | | | Imputed data | | |
| --- | --- | --- | --- | --- | --- | --- | --- |
|  |  | % | 95% CI | | % | 95% CI | |
|  |  |  | lo | hi |  | lo | hi |
| Cannabis use | |  |  |  |  |  |  |
|  | Daily | 1.5% | 1.5% | 1.6% | 1.5% | 1.5% | 1.6% |
|  | Weekly | 2.2% | 2.1% | 2.4% | 2.3% | 2.1% | 2.4% |
|  | Less than weekly | 6.7% | 6.5% | 6.9% | 6.7% | 6.5% | 6.9% |
|  | No use | 89.5% | 89.3% | 89.8% | 89.5% | 89.3% | 89.7% |
| Sex |  |  |  |  |  |  |  |
|  | Male | 49.4% | 49.0% | 49.7% | 49.4% | 49.0% | 49.7% |
|  | Female | 50.6% | 50.3% | 51.0% | 50.6% | 50.3% | 51.0% |
| Age |  |  |  |  |  |  |  |
|  | 12-17 | 8.4% | 8.2% | 8.7% | -- | -- | -- |
|  | 18-24 | 11.7% | 11.5% | 12.0% | -- | -- | -- |
|  | 25-29 | 8.3% | 8.1% | 8.5% | -- | -- | -- |
|  | 30-39 | 17.4% | 17.1% | 17.7% | -- | -- | -- |
|  | 40-49 | 16.8% | 16.5% | 17.0% | -- | -- | -- |
|  | 50-59 | 14.9% | 14.7% | 15.2% | -- | -- | -- |
|  | 60-69 | 11.8% | 11.6% | 12.0% | -- | -- | -- |
|  | 70+ | 10.6% | 10.4% | 10.8% | -- | -- | -- |
| Area of residence | |  |  |  |  |  |  |
|  | Major cities | 72.8% | 72.5% | 73.1% | -- | -- | -- |
|  | Inner regional | 17.4% | 17.2% | 17.7% | -- | -- | -- |
|  | Outer regional or rural | 9.8% | 9.6% | 9.9% | -- | -- | -- |
| Marital status | |  |  |  |  |  |  |
|  | Not partnered | 39.5% | 39.2% | 39.9% | 40.2% | 39.8% | 40.6% |
|  | Married/defacto | 60.5% | 60.1% | 60.8% | 59.8% | 59.4% | 60.2% |
| Employment status | |  |  |  |  |  |  |
|  | Not employed | 41.3% | 40.9% | 41.6% | 42.0% | 41.6% | 42.3% |
|  | Employed | 58.7% | 58.4% | 59.1% | 58.0% | 57.7% | 58.4% |
| Education | |  |  |  |  |  |  |
|  | No high school | 35.1% | 34.8% | 35.5% | 35.0% | 34.7% | 35.4% |
|  | Completed high school | 64.9% | 64.5% | 65.2% | 65.0% | 64.6% | 65.3% |
| SEIFA quintile | |  |  |  |  |  |  |
|  | Lowest | 16.9% | 16.7% | 17.2% | -- | -- | -- |
|  | 2nd | 19.5% | 19.3% | 19.8% | -- | -- | -- |
|  | 3rd | 19.6% | 19.3% | 19.8% | -- | -- | -- |
|  | 4th | 20.6% | 20.4% | 20.9% | -- | -- | -- |
|  | Highest | 23.3% | 23.0% | 23.6% | -- | -- | -- |
| Note. There was no missing data for age, area of residence, and SEIFA therefore no imputations were required; SEIFA=Socio-Economic Indexes for Areas. | | | | | | | |

**Table S3. Data table for weighted frequency of cannabis use by year in the NDSHS 2001-2022/23 (n=197,484)**

| Cannabis use frequency by year | | Original data | | | Imputed data | | |
| --- | --- | --- | --- | --- | --- | --- | --- |
|  |  | % | 95% CI | | % | 95% CI | |
|  |  |  | lo | hi |  | lo | hi |
| 2001 | |  |  |  |  |  |  |
|  | Daily | 1.67% | 1.41% | 1.98% | 1.67% | 1.39% | 1.96% |
|  | Weekly | 2.74% | 2.38% | 3.15% | 2.74% | 2.35% | 3.13% |
|  | Less than weekly | 7.40% | 6.80% | 8.04% | 7.36% | 6.74% | 7.98% |
|  | No use | 88.19% | 87.41% | 88.93% | 88.22% | 87.46% | 88.99% |
| 2004 | |  |  |  |  |  |  |
|  | Daily | 1.62% | 1.41% | 1.85% | 1.62% | 1.40% | 1.83% |
|  | Weekly | 2.45% | 2.18% | 2.74% | 2.44% | 2.16% | 2.72% |
|  | Less than weekly | 6.78% | 6.32% | 7.26% | 6.77% | 6.30% | 7.23% |
|  | No use | 89.16% | 88.58% | 89.72% | 89.18% | 88.61% | 89.75% |
| 2007 | |  |  |  |  |  |  |
|  | Daily | 1.25% | 1.01% | 1.53% | 1.26% | 1.00% | 1.52% |
|  | Weekly | 1.52% | 1.28% | 1.80% | 1.54% | 1.27% | 1.80% |
|  | Less than weekly | 5.89% | 5.30% | 6.53% | 5.90% | 5.29% | 6.51% |
|  | No use | 91.35% | 90.63% | 92.02% | 91.31% | 90.61% | 92.00% |
| 2010 | |  |  |  |  |  |  |
|  | Daily | 1.29% | 1.13% | 1.48% | 1.31% | 1.13% | 1.49% |
|  | Weekly | 2.08% | 1.88% | 2.29% | 2.08% | 1.87% | 2.29% |
|  | Less than weekly | 6.57% | 6.20% | 6.96% | 6.58% | 6.20% | 6.97% |
|  | No use | 90.07% | 89.60% | 90.51% | 90.02% | 89.57% | 90.48% |
| 2013 | |  |  |  |  |  |  |
|  | Daily | 1.25% | 1.08% | 1.45% | 1.24% | 1.06% | 1.43% |
|  | Weekly | 1.89% | 1.69% | 2.12% | 1.90% | 1.68% | 2.12% |
|  | Less than weekly | 6.58% | 6.19% | 6.99% | 6.58% | 6.18% | 6.98% |
|  | No use | 90.28% | 89.79% | 90.75% | 90.28% | 89.81% | 90.76% |
| 2016 | |  |  |  |  |  |  |
|  | Daily | 1.45% | 1.26% | 1.67% | 1.45% | 1.24% | 1.66% |
|  | Weekly but less than daily | 2.23% | 1.99% | 2.49% | 2.23% | 1.98% | 2.48% |
|  | Less than weekly | 6.42% | 6.04% | 6.83% | 6.42% | 6.03% | 6.82% |
|  | No use | 89.90% | 89.40% | 90.38% | 89.90% | 89.40% | 90.39% |
| 2019 | |  |  |  |  |  |  |
|  | Daily | 1.62% | 1.42% | 1.85% | 1.62% | 1.41% | 1.83% |
|  | Weekly but less than daily | 2.53% | 2.25% | 2.85% | 2.53% | 2.24% | 2.83% |
|  | Less than weekly | 7.04% | 6.61% | 7.49% | 7.02% | 6.58% | 7.47% |
|  | No use | 88.81% | 88.25% | 89.35% | 88.82% | 88.27% | 89.38% |
| 2022/23 | |  |  |  |  |  |  |
|  | Daily | 2.17% | 1.92% | 2.46% | 2.16% | 1.89% | 2.42% |
|  | Weekly but less than daily | 2.37% | 2.12% | 2.66% | 2.36% | 2.09% | 2.63% |
|  | Less than weekly | 6.66% | 6.23% | 7.13% | 6.65% | 6.20% | 7.09% |
|  | No use | 88.79% | 88.22% | 89.35% | 88.83% | 88.27% | 89.40% |

**Table S4. Prevalence of cannabis use by sociodemographic factors in the NDSHS 2001-2022/23 combined (N=197,484)**

| NDSHS 2001-2022-23 combined | | Weighted prevalence of cannabis use with 95% confidence intervals | | | | | | | | | | | |
| --- | --- | --- | --- | --- | --- | --- | --- | --- | --- | --- | --- | --- | --- |
|  |  | Daily | | | Weekly but less than daily | | | Less than weekly | | | No use | | |
|  |  | % | lo | hi | % | lo | hi | % | lo | hi | % | lo | hi |
| Sex |  |  |  |  |  |  |  |  |  |  |  |  |  |
|  | Male | 2.12% | 1.96% | 2.28% | 3.15% | 2.95% | 3.36% | 7.97% | 7.65% | 8.30% | 86.75% | 86.35% | 87.15% |
|  | Female | 0.99% | 0.90% | 1.07% | 1.38% | 1.27% | 1.48% | 5.47% | 5.24% | 5.69% | 92.17% | 91.91% | 92.43% |
| Age |  |  |  |  |  |  |  |  |  |  |  |  |  |
|  | 12-17 | 1.03% | 0.65% | 1.41% | 1.98% | 1.53% | 2.44% | 7.64% | 6.85% | 8.43% | 89.34% | 88.38% | 90.30% |
|  | 18-24 | 2.92% | 2.54% | 3.31% | 5.07% | 4.52% | 5.62% | 16.94% | 15.96% | 17.92% | 75.06% | 73.95% | 76.17% |
|  | 25-29 | 3.15% | 2.67% | 3.62% | 4.19% | 3.62% | 4.75% | 13.25% | 12.37% | 14.13% | 79.41% | 78.32% | 80.51% |
|  | 30-39 | 2.11% | 1.88% | 2.34% | 2.99% | 2.70% | 3.28% | 8.54% | 8.02% | 9.06% | 86.36% | 85.75% | 86.97% |
|  | 40-49 | 1.76% | 1.54% | 1.99% | 1.91% | 1.70% | 2.12% | 5.27% | 4.90% | 5.63% | 91.05% | 90.59% | 91.52% |
|  | 50-59 | 0.94% | 0.80% | 1.08% | 1.47% | 1.28% | 1.66% | 2.82% | 2.58% | 3.06% | 94.77% | 94.43% | 95.10% |
|  | 60-69 | 0.34% | 0.28% | 0.41% | 0.61% | 0.49% | 0.73% | 1.30% | 1.04% | 1.55% | 97.75% | 97.46% | 98.03% |
|  | 70+ | 0.12% | 0.05% | 0.19% | 0.08% | 0.04% | 0.13% | 0.25% | 0.17% | 0.34% | 99.55% | 99.43% | 99.66% |
| Area of residence | |  |  |  |  |  |  |  |  |  |  |  |  |
|  | Major cities | 1.43% | 1.32% | 1.53% | 2.21% | 2.07% | 2.34% | 7.01% | 6.76% | 7.25% | 89.36% | 89.07% | 89.65% |
|  | Inner regional | 1.69% | 1.47% | 1.91% | 2.32% | 2.05% | 2.59% | 5.88% | 5.45% | 6.30% | 90.11% | 89.58% | 90.64% |
|  | Outer regional or rural | 2.19% | 1.88% | 2.51% | 2.47% | 2.18% | 2.76% | 5.95% | 5.47% | 6.43% | 89.39% | 88.76% | 90.01% |
| Marital status | |  |  |  |  |  |  |  |  |  |  |  |  |
|  | Not partnered | 2.40% | 2.21% | 2.58% | 3.59% | 3.35% | 3.82% | 10.68% | 10.28% | 11.09% | 83.33% | 82.85% | 83.81% |
|  | Married/defacto | 0.98% | 0.89% | 1.07% | 1.36% | 1.25% | 1.46% | 4.03% | 3.85% | 4.21% | 93.64% | 93.42% | 93.86% |
| Employment status | |  |  |  |  |  |  |  |  |  |  |  |  |
|  | Not employed | 1.34% | 1.21% | 1.47% | 1.72% | 1.57% | 1.87% | 4.79% | 4.51% | 5.06% | 92.15% | 91.82% | 92.48% |
|  | Employed | 1.70% | 1.57% | 1.82% | 2.64% | 2.47% | 2.80% | 8.09% | 7.81% | 8.37% | 87.58% | 87.24% | 87.91% |
| Education | |  |  |  |  |  |  |  |  |  |  |  |  |
|  | No high school | 1.78% | 1.61% | 1.95% | 2.19% | 1.99% | 2.38% | 4.82% | 4.52% | 5.11% | 91.22% | 90.83% | 91.60% |
|  | Completed high school | 1.42% | 1.32% | 1.53% | 2.29% | 2.15% | 2.43% | 7.72% | 7.46% | 7.98% | 88.57% | 88.26% | 88.87% |
| SEIFA quintile | |  |  |  |  |  |  |  |  |  |  |  |  |
|  | Lowest | 2.29% | 2.02% | 2.56% | 2.69% | 2.40% | 2.99% | 5.47% | 5.06% | 5.87% | 89.55% | 89.00% | 90.10% |
|  | 2nd | 1.79% | 1.59% | 1.98% | 2.18% | 1.95% | 2.40% | 5.88% | 5.50% | 6.25% | 90.16% | 89.69% | 90.63% |
|  | 3rd | 1.58% | 1.37% | 1.80% | 2.28% | 2.04% | 2.53% | 6.70% | 6.26% | 7.13% | 89.44% | 88.92% | 89.96% |
|  | 4th | 1.29% | 1.09% | 1.49% | 2.16% | 1.90% | 2.42% | 7.01% | 6.55% | 7.47% | 89.54% | 88.99% | 90.08% |
|  | Highest | 1.01% | 0.85% | 1.17% | 2.06% | 1.80% | 2.31% | 8.04% | 7.56% | 8.52% | 88.89% | 88.34% | 89.44% |

Note. Missing data were imputed using multiple imputations.

**Table S5. Significant year interactions from the multinomial regression on cannabis use**

| Year x Age interactions were significant and presented | | | Adjusted odds ratios (ref=no use) | | | | | |
| --- | --- | --- | --- | --- | --- | --- | --- | --- |
|  |  |  | Daily use | | Weekly but less than daily | | Less than weekly | |
|  |  |  | vs no use |  | vs no use |  | vs no use |  |
|  |  |  | OR [95%CI] | p | OR [95%CI] | p | OR [95%CI] | p |
| 2004 | x | 12-17 | 0.15 [0.08-0.30] | <0.001 | 0.27 [0.17-0.44] | <0.001 | 0.51 [0.38-0.70] | <0.001 |
| 2004 | x | 18-24 | 0.44 [0.29-0.67] | <0.001 | 0.60 [0.42-0.86] | 0.005 | 0.90 [0.71-1.14] | 0.387 |
| 2004 | x | 25-29 | 0.76 [0.50-1.16] | 0.205 | 0.62 [0.43-0.90] | 0.013 | 0.99 [0.77-1.26] | 0.913 |
| 2004 | x | 30-39 | 0.89 [0.61-1.31] | 0.562 | 0.65 [0.47-0.89] | 0.007 | 0.94 [0.76-1.17] | 0.595 |
| 2004 | x | 50-59 | 0.89 [0.44-1.79] | 0.750 | 1.01 [0.60-1.70] | 0.981 | 0.98 [0.70-1.36] | 0.889 |
| 2004 | x | 60+ | nr |  | 0.75 [0.21-2.64] | 0.651 | 0.96 [0.46-2.01] | 0.916 |
| 2007 | x | 12-17 | 0.08 [0.03-0.20] | <0.001 | 0.14 [0.07-0.27] | <0.001 | 0.51 [0.35-0.73] | <0.001 |
| 2007 | x | 18-24 | 0.25 [0.15-0.42] | <0.001 | 0.36 [0.24-0.55] | <0.001 | 0.80 [0.61-1.04] | 0.101 |
| 2007 | x | 25-29 | 0.57 [0.35-0.92] | 0.021 | 0.51 [0.33-0.78] | 0.002 | 0.85 [0.65-1.12] | 0.260 |
| 2007 | x | 30-39 | 0.64 [0.42-0.97] | 0.036 | 0.47 [0.33-0.68] | <0.001 | 0.88 [0.69-1.13] | 0.332 |
| 2007 | x | 50-59 | 0.99 [0.48-2.06] | 0.980 | 1.35 [0.79-2.31] | 0.277 | 1.11 [0.78-1.58] | 0.572 |
| 2007 | x | 60+ | 3619.74 [0.00-.] | 0.982 | 1.07 [0.32-3.58] | 0.907 | 1.66 [0.83-3.33] | 0.154 |
| 2010 | x | 12-17 | 0.06 [0.02-0.18] | <0.001 | 0.16 [0.09-0.30] | <0.001 | 0.40 [0.28-0.55] | <0.001 |
| 2010 | x | 18-24 | 0.27 [0.16-0.44] | <0.001 | 0.39 [0.26-0.57] | <0.001 | 0.64 [0.49-0.82] | <0.001 |
| 2010 | x | 25-29 | 0.37 [0.23-0.60] | <0.001 | 0.50 [0.33-0.75] | 0.001 | 0.69 [0.54-0.90] | 0.005 |
| 2010 | x | 30-39 | 0.50 [0.33-0.76] | 0.001 | 0.56 [0.40-0.79] | 0.001 | 0.83 [0.67-1.04] | 0.114 |
| 2010 | x | 50-59 | 2.18 [1.15-4.14] | 0.017 | 2.26 [1.38-3.70] | 0.001 | 1.23 [0.89-1.69] | 0.206 |
| 2010 | x | 60+ | 3721.53 [0.00-.] | 0.982 | 1.61 [0.55-4.66] | 0.383 | 1.32 [0.67-2.61] | 0.422 |
| 2013 | x | 12-17 | 0.17 [0.08-0.39] | <0.001 | 0.22 [0.12-0.41] | <0.001 | 0.34 [0.23-0.49] | <0.001 |
| 2013 | x | 18-24 | 0.27 [0.17-0.45] | <0.001 | 0.46 [0.31-0.70] | <0.001 | 0.57 [0.44-0.74] | <0.001 |
| 2013 | x | 25-29 | 0.30 [0.18-0.51] | <0.001 | 0.35 [0.22-0.55] | <0.001 | 0.62 [0.47-0.80] | <0.001 |
| 2013 | x | 30-39 | 0.40 [0.25-0.62] | <0.001 | 0.50 [0.34-0.72] | <0.001 | 0.73 [0.58-0.92] | 0.008 |
| 2013 | x | 50-59 | 2.51 [1.32-4.79] | 0.005 | 2.28 [1.36-3.82] | 0.002 | 1.63 [1.20-2.24] | 0.002 |
| 2013 | x | 60+ | 5071.61 [0.00-.] | 0.982 | 3.86 [1.41-10.59] | 0.009 | 2.28 [1.23-4.21] | 0.009 |
| 2016 | x | 12-17 | 0.07 [0.03-0.20] | <0.001 | 0.17 [0.09-0.33] | <0.001 | 0.24 [0.16-0.36] | <0.001 |
| 2016 | x | 18-24 | 0.28 [0.17-0.45] | <0.001 | 0.44 [0.29-0.67] | <0.001 | 0.47 [0.36-0.61] | <0.001 |
| 2016 | x | 25-29 | 0.30 [0.18-0.49] | <0.001 | 0.42 [0.27-0.66] | <0.001 | 0.52 [0.40-0.67] | <0.001 |
| 2016 | x | 30-39 | 0.41 [0.26-0.64] | <0.001 | 0.46 [0.32-0.66] | <0.001 | 0.65 [0.51-0.81] | <0.001 |
| 2016 | x | 50-59 | 2.32 [1.21-4.44] | 0.011 | 2.63 [1.58-4.35] | <0.001 | 1.53 [1.12-2.08] | 0.008 |
| 2016 | x | 60+ | nr |  | 4.86 [1.77-13.34] | 0.002 | 4.10 [2.26-7.46] | <0.001 |
| 2019 | x | 12-17 | 0.12 [0.04-0.38] | <0.001 | 0.36 [0.17-0.76] | 0.007 | 0.32 [0.21-0.50] | <0.001 |
| 2019 | x | 18-24 | 0.19 [0.12-0.33] | <0.001 | 0.52 [0.34-0.80] | 0.003 | 0.55 [0.42-0.71] | <0.001 |
| 2019 | x | 25-29 | 0.26 [0.15-0.44] | <0.001 | 0.51 [0.33-0.80] | 0.003 | 0.60 [0.46-0.78] | <0.001 |
| 2019 | x | 30-39 | 0.45 [0.29-0.70] | <0.001 | 0.41 [0.28-0.61] | <0.001 | 0.66 [0.52-0.83] | <0.001 |
| 2019 | x | 50-59 | 3.36 [1.77-6.35] | <0.001 | 4.05 [2.45-6.70] | <0.001 | 1.80 [1.32-2.45] | <0.001 |
| 2019 | x | 60+ | nr |  | 9.01 [3.36-24.12] | <0.001 | 4.58 [2.53-8.26] | <0.001 |
| 2022/23 | x | 12-17 | 0.16 [0.06-0.40] | <0.001 | 0.38 [0.18-0.78] | 0.008 | 0.38 [0.24-0.60] | <0.001 |
| 2022/23 | x | 18-24 | 0.24 [0.15-0.39] | <0.001 | 0.45 [0.28-0.71] | 0.001 | 0.62 [0.47-0.82] | 0.001 |
| 2022/23 | x | 25-29 | 0.22 [0.13-0.36] | <0.001 | 0.50 [0.32-0.79] | 0.003 | 0.61 [0.46-0.81] | 0.001 |
| 2022/23 | x | 30-39 | 0.34 [0.22-0.53] | <0.001 | 0.63 [0.43-0.92] | 0.016 | 0.77 [0.61-0.97] | 0.029 |
| 2022/23 | x | 50-59 | 3.31 [1.77-6.22] | <0.001 | 3.96 [2.37-6.63] | <0.001 | 1.79 [1.30-2.45] | <0.001 |
| 2022/23 | x | 60+ | nr |  | 13.21 [4.86-35.92] | <0.001 | 6.36 [3.49-11.60] | <0.001 |
| Model included year, sex, age, area of residence, marital status, employment status, education, SEIFA. The model included main effects and interaction terms between each of the variable and year. SEIFA: Socio-Economic Indexes for Areas. All variables were entered into the model together and ORs presented are adjusted odds ratios. A p-value of <0.001 was used to indicate significance. nr: not reported due to small cell size. Only variables with significant year interaction was presented in the table. | | | | | | | | |

**Table S6. Prevalence of cannabis use by age in the NDSHS 2001-2022/23**

| Cannabis use by age | | Weighted prevalence of daily cannabis use and 95% confidence intervals | | | | | | | | | | | |
| --- | --- | --- | --- | --- | --- | --- | --- | --- | --- | --- | --- | --- | --- |
|  |  | 2001 | | | 2004 | | | 2007 | | | 2010 | | |
|  |  | % | lo | hi | % | lo | hi | % | lo | hi | % | lo | hi |
| **Daily cannabis use** | |  |  |  |  |  |  |  |  |  |  |  |  |
|  | 12-17 | 3.07% | 1.22% | 4.91% | 0.56% | 0.28% | 0.85% | 0.37% | -0.03% | 0.77% | -0.06% | 1.22% | 0.45% |
|  | 18-24 | 3.29% | 2.36% | 4.21% | 3.08% | 2.14% | 4.22% | 2.46% | 1.17% | 3.74% | 2.52% | 1.64% | 3.39% |
|  | 25-29 | 3.64% | 2.17% | 5.10% | 3.77% | 2.75% | 4.78% | 3.54% | 1.89% | 5.18% | 2.71% | 1.82% | 3.61% |
|  | 30-39 | 2.37% | 1.66% | 3.07% | 2.98% | 2.24% | 3.72% | 1.70% | 1.16% | 2.23% | 1.89% | 1.39% | 2.39% |
|  | 40-49 | 1.47% | 0.84% | 2.09% | 1.74% | 1.20% | 2.29% | 1.56% | 0.92% | 2.20% | 1.70% | 1.26% | 2.14% |
|  | 50-59 | 0.17% | 0.06% | 0.27% | 0.35% | 0.16% | 0.54% | 0.69% | 0.11% | 1.26% | 0.91% | 0.62% | 1.21% |
|  | 60-69 | -- | -- | -- | 0.04% | 0.00% | 0.08% | 0.07% | 0.00% | 0.14% | 0.18% | 0.01% | 0.35% |
|  | 70+ | -- | -- | -- | -- | -- | -- | -- | -- | -- | -- | -- | -- |
|  | | 2013 | | | 2016 | | | 2019 | | | 2022/23 | | |
|  |  | % | lo | hi | % | lo | hi | % | lo | hi | % | lo | hi |
|  | 12-17 | 1.04% | 0.12% | 1.96% | 0.52% | 0.00% | 1.06% | 0.45% | 0.00% | 0.98% | 1.48% | 0.28% | 2.68% |
|  | 18-24 | 2.42% | 1.59% | 3.24% | 3.19% | 2.10% | 4.27% | 2.41% | 1.50% | 3.33% | 3.81% | 2.56% | 5.06% |
|  | 25-29 | 1.95% | 1.23% | 2.67% | 2.81% | 1.69% | 3.94% | 2.39% | 1.44% | 3.33% | 3.00% | 1.83% | 4.16% |
|  | 30-39 | 1.36% | 0.94% | 1.78% | 1.63% | 1.17% | 2.10% | 2.05% | 1.52% | 2.57% | 2.23% | 1.60% | 2.87% |
|  | 40-49 | 1.54% | 1.12% | 1.97% | 1.66% | 1.22% | 2.11% | 2.36% | 1.70% | 3.03% | 2.57% | 1.96% | 3.19% |
|  | 50-59 | 1.27% | 0.89% | 1.65% | 1.26% | 0.87% | 1.65% | 1.68% | 1.21% | 2.15% | 2.53% | 1.86% | 3.20% |
|  | 60-69 | 0.29% | 0.05% | 0.53% | 0.56% | 0.30% | 0.81% | 0.71% | 0.40% | 1.02% | 1.38% | 1.00% | 1.76% |
|  | 70+ | -- | -- | -- | 0.12% | -0.02% | 0.26% | 0.29% | 0.06% | 0.52% | 0.54% | 0.08% | 1.00% |
| **Weekly cannabis use (but not daily)** | | | |  |  |  |  |  |  |  |  |  |  |
|  | | 2001 | | | 2004 | | | 2007 | | | 2010 | | |
|  |  | % | lo | hi | % | lo | hi | % | lo | hi | % | lo | hi |
|  |  |  |  |  |  |  |  |  |  |  |  |  |  |
|  | 12-17 | 4.66% | 2.46% | 6.86% | 1.84% | 1.32% | 2.36% | 0.72% | 0.29% | 1.16% | 0.99% | 0.55% | 1.48% |
|  | 18-24 | 6.97% | 5.07% | 8.86% | 5.73% | 4.42% | 7.03% | 3.06% | 1.71% | 4.40% | 4.00% | 3.06% | 4.95% |
|  | 25-29 | 5.77% | 3.82% | 7.72% | 5.40% | 3.68% | 7.11% | 2.74% | 1.70% | 3.78% | 3.68% | 2.67% | 4.69% |
|  | 30-39 | 3.73% | 2.79% | 4.67% | 3.50% | 2.75% | 4.25% | 2.17% | 1.50% | 2.84% | 3.14% | 2.53% | 3.74% |
|  | 40-49 | 1.50% | 1.02% | 1.98% | 1.99% | 1.51% | 2.46% | 1.85% | 1.13% | 2.57% | 2.21% | 1.75% | 2.68% |
|  | 50-59 | 0.65% | 0.25% | 1.05% | 0.87% | 0.43% | 1.31% | 1.12% | 0.63% | 1.60% | 1.84% | 1.38% | 2.30% |
|  | 60-69 | 0.03% | -0.01% | 0.07% | 0.14% | 0.01% | 0.27% | 0.32% | 0.00% | 0.67% | 0.22% | 0.09% | 0.34% |
|  | 70+ | 0.02% | -0.01% | 0.04% | -- | -- | -- | -- | -- | -- |  |  |  |
|  |  | 2013 | | | 2016 | | | 2019 | | | 2022/23 | | |
|  |  | % | lo | hi | % | lo | hi | % | lo | hi | % | lo | hi |
|  | 12-17 | 1.69% | 0.91% | 2.48% | 1.13% | 0.44% | 1.81% | 1.85% | 0.65% | 3.06% | 2.31% | 0.86% | 3.76% |
|  | 18-24 | 4.23% | 3.10% | 5.35% | 5.30% | 4.00% | 6.59% | 5.63% | 4.18% | 7.08% | 4.55% | 3.11% | 5.99% |
|  | 25-29 | 2.64% | 1.73% | 3.55% | 3.55% | 2.43% | 4.67% | 4.33% | 2.91% | 5.76% | 3.35% | 2.26% | 4.43% |
|  | 30-39 | 2.51% | 1.93% | 3.09% | 2.80% | 2.13% | 3.46% | 2.32% | 1.72% | 2.91% | 3.01% | 2.36% | 3.67% |
|  | 40-49 | 1.76% | 1.33% | 2.19% | 2.34% | 1.77% | 2.92% | 2.25% | 1.59% | 2.91% | 1.95% | 1.40% | 2.49% |
|  | 50-59 | 1.65% | 1.20% | 2.11% | 1.96% | 1.43% | 2.50% | 2.80% | 2.15% | 3.46% | 2.31% | 1.74% | 2.88% |
|  | 60-69 | 0.59% | 0.36% | 0.83% | 0.68% | 0.44% | 0.92% | 1.60% | 0.96% | 2.23% | 1.93% | 1.40% | 2.45% |
|  | 70+ | -- | -- | -- | 0.16% | 0.00% | 0.36% | 0.15% | 0.00% | 0.29% | 0.33% | 0.13% | 0.54% |
| **Less than weekly cannabis use (use but less than weekly)** | | | | |  |  |  |  |  |  |  |  |  |
|  | | 2001 | | | 2004 | | | 2007 | | | 2010 | | |
|  |  | % | lo | hi | % | lo | hi | % | lo | hi | % | lo | hi |
|  |  |  |  |  |  |  |  |  |  |  |  |  |  |
|  | 12-17 | 13.29% | 10.21% | 16.37% | 7.61% | 6.51% | 8.71% | 6.42% | 4.33% | 8.51% | 7.76% | 6.18% | 9.34% |
|  | 18-24 | 19.18% | 16.42% | 21.94% | 17.95% | 15.64% | 20.25% | 16.06% | 12.77% | 19.36% | 15.88% | 14.05% | 17.72% |
|  | 25-29 | 13.72% | 11.05% | 16.38% | 13.38% | 11.25% | 15.51% | 11.56% | 8.94% | 14.17% | 13.41% | 11.55% | 15.27% |
|  | 30-39 | 9.68% | 7.99% | 11.36% | 8.76% | 7.49% | 10.03% | 7.22% | 5.84% | 8.59% | 8.45% | 7.55% | 9.36% |
|  | 40-49 | 4.04% | 3.12% | 4.95% | 4.64% | 3.74% | 5.53% | 4.86% | 3.65% | 6.07% | 5.46% | 4.70% | 6.21% |
|  | 50-59 | 1.65% | 0.99% | 2.31% | 1.70% | 1.28% | 2.12% | 2.17% | 1.44% | 2.90% | 2.69% | 2.17% | 3.22% |
|  | 60-69 | 1.12% | -0.05% | 2.30% | 0.37% | 0.09% | 0.65% | 0.71% | 0.22% | 1.21% | 0.57% | 0.31% | 0.84% |
|  | 70+ | 0.02% | -0.02% | 0.05% | -- | -- | -- | -- | -- | -- | -- | -- | -- |
|  |  | 2013 | | | 2016 | | | 2019 | | | 2022/23 | | |
|  |  | % | lo | hi | % | lo | hi | % | lo | hi | % | lo | hi |
|  | 12-17 | 6.06% | 4.57% | 7.56% | 3.83% | 2.66% | 5.01% | 5.75% | 3.82% | 7.68% | 5.38% | 3.36% | 7.39% |
|  | 18-24 | 16.12% | 14.11% | 18.14% | 14.71% | 12.72% | 16.69% | 16.07% | 13.91% | 18.22% | 15.75% | 13.31% | 18.19% |
|  | 25-29 | 12.94% | 11.07% | 14.82% | 13.07% | 11.12% | 15.02% | 14.05% | 11.90% | 16.19% | 13.03% | 10.82% | 15.25% |
|  | 30-39 | 8.20% | 7.24% | 9.16% | 8.24% | 7.26% | 9.22% | 8.58% | 7.51% | 9.66% | 8.25% | 7.21% | 9.28% |
|  | 40-49 | 5.97% | 5.14% | 6.79% | 6.68% | 5.78% | 7.59% | 6.51% | 5.58% | 7.43% | 6.57% | 5.55% | 7.59% |
|  | 50-59 | 4.01% | 3.32% | 4.69% | 3.96% | 3.28% | 4.65% | 4.68% | 3.88% | 5.48% | 4.33% | 3.51% | 5.15% |
|  | 60-69 | 0.92% | 0.60% | 1.24% | 2.01% | 1.52% | 2.49% | 2.43% | 1.84% | 3.02% | 2.96% | 2.32% | 3.59% |
|  | 70+ | 0.15% | 0.02% | 0.27% | 0.45% | 0.18% | 0.72% | 0.61% | 0.24% | 0.99% | 0.76% | 0.44% | 1.08% |
